# Lymphoscintigraphy and triangulated body marking for morbidity reduction during sentinel node biopsy in breast cancer

**DOI:** 10.1186/1477-7800-2-25

**Published:** 2005-11-08

**Authors:** Borys R Krynyckyi, Michail K Shafir, Suk Chul Kim, Dong Wook Kim, Arlene Travis, Renee M Moadel, Chun K Kim

**Affiliations:** 1 Department of Radiology, Division of Nuclear Medicine, The Mount Sinai School of Medicine, The Mount Sinai Hospital, New York, New York, USA; 2 Department of Surgery, The Mount Sinai School of Medicine, The Mount Sinai Hospital, New York, New York, USA; 3 Department of Nuclear Medicine, Albert Einstein College of Medicine of Yeshiva University, and the Montefiore Medical Center, Bronx, New York, USA

## Abstract

Current trends in patient care include the desire for minimizing invasiveness of procedures and interventions. This aim is reflected in the increasing utilization of sentinel lymph node biopsy, which results in a lower level of morbidity in breast cancer staging, in comparison to extensive conventional axillary dissection. Optimized lymphoscintigraphy with triangulated body marking is a clinical option that can further reduce morbidity, more than when a hand held gamma probe alone is utilized. Unfortunately it is often either overlooked or not fully understood, and thus not utilized. This results in the unnecessary loss of an opportunity to further reduce morbidity.

Optimized lymphoscintigraphy and triangulated body marking provides a detailed 3 dimensional map of the number and location of the sentinel nodes, available before the first incision is made. The number, location, relevance based on time/sequence of appearance of the nodes, all can influence 1) where the incision is made, 2) how extensive the dissection is, and 3) how many nodes are removed. In addition, complex patterns can arise from injections. These include prominent lymphatic channels, pseudo-sentinel nodes, echelon and reverse echelon nodes and even contamination, which are much more difficult to access with the probe only. With the detailed information provided by optimized lymphoscintigraphy and triangulated body marking, the surgeon can approach the axilla in a more enlightened fashion, in contrast to when the less informed probe only method is used. This allows for better planning, resulting in the best cosmetic effect and less trauma to the tissues, further reducing morbidity while maintaining adequate sampling of the sentinel node(s).

## Introduction

The only goal of sentinel lymph node biopsy (SLNB) is to prevent/reduce morbidity associated with axillary lymph node dissection (ALND) while maintaining or enhancing sensitivity for detecting nodal disease. It has been extensively demonstrated that SLNB is associated with lower morbidity than ALND [1-24]. Lymphoscintigraphy potentially offers further reductions in morbidity, as compared to a probe-only methodology.

**Figure 1:**
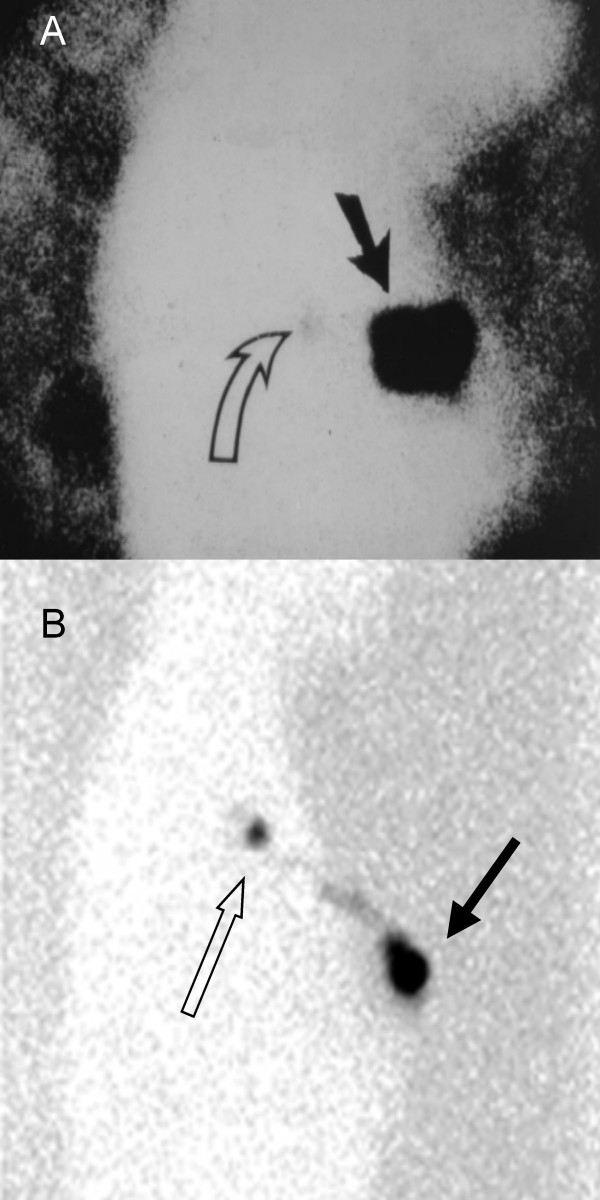
(A*) TOP: Labeled as a "typical" lymphoscintigraphy finding in an article disputing the value of lymphoscintigraphy, the lateral view depicts the poor quality of injection and imaging technique [37]. The injection site is represented by the solid arrow, the faint, barely visible SN by the open arrow. (B) BOTTOM: Right lateral view of typical/average result from optimized injection and imaging protocol showing injection sites (solid arrow) and bright sentinel node (open arrow) as well as lymphatic channel leading to sentinel node [38]. In many cases, even much brighter nodes than depicted in B are found. *Reprinted from Am J Surg. 177, Burak WE Jr, Routine preoperative lymphoscintigraphy is not necessary prior to sentinel node biopsy for breast cancer, 445–449., 1999, with permission from Elsevier Ltd.; Excerpta Medica Inc . [37].

**Figure 2:**
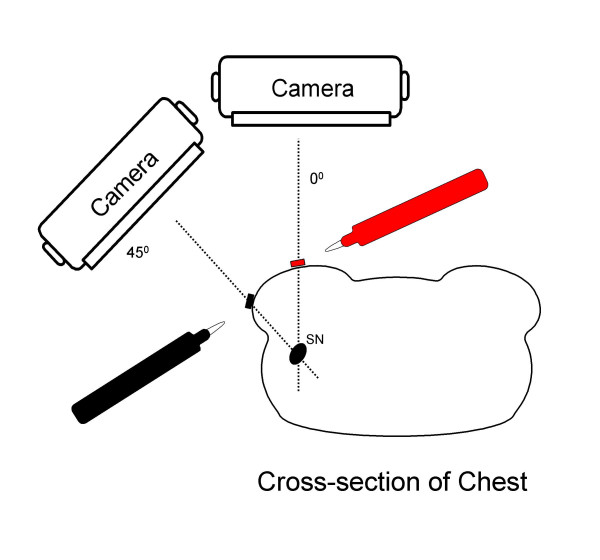
Schematic of triangulated patient body marking technique. Different colored permanent markers are used to place reference points on the patient's body corresponding to the location of a sentinel node along a particular projection. With this form of triangulation, the location of the sentinel nodes can be defined in 3 dimensions along appropriate triangulation lines. The arm is maintained in the surgical position (90°) to eliminate shifting of skin markings*. The rotation of the torso referenced to the floor must be kept constant during both imaging and surgery for the relationships to remain valid, or compensated for by equally shifted projections if rotation is desired during surgery [40]. *Adapted, revised and used with permission from *Radiographics *. 2004;24:121–145. Krynyckyi BR, et al. RSNA Publications, Oak Brook, IL. [ref. 38].

Some surgeons do not utilize lymphoscintigraphy at the time of SLNB and controversies continue [11,25-36]. One factor that adds confusion to understanding the value of lymphoscintigraphy is that when it is performed, there is often wide variation in techniques and quality [37-40], (figure 1). However, when optimized lymphoscintigraphy with triangulated patient body marking is not performed, an opportunity to further reduce morbidity is missed [38,40,41], [figure 2, table 1, 2, 3]. Below is a discussion of the advantages of lymphoscintigraphy, in which we seek to clarify misconceptions about its utility, discuss optimal techniques, provide an updated review of literature available on the added benefits of lymphoscintigraphy, and discuss its value in special situations.

**Table 1:** Comparisons of average chronic pain and numbness/paresthesia morbidity between LS groups (+) performing lymphoscintigraphy and non LS groups (-) not performing lymphoscintigraphy in patients undergoing SLNB using radiotracer or using only dye. In general, studies using lymphoscintigraphy have much lower levels of chronic sensory morbidity. Data from original reference by Kim SC et al. [41].

	Lymphoscintigraphy (+) Performed			Lymphoscintigraphy (-) Not Performed			
		
*Morbidity (Mor)	Mor (%)	Total Pt (N)	References	Mor (%)	Total Pt (N)	References	^#^p-value
Pain (>9m)	13.77%	1365	1,2,4,9,10,11,14,20	28.67%	143	6	< 0.0001
Numbness/Paresthesia (>9m)	12.56%	677	1,4,9,11,13,14,17,20	23.14%	229	3,6,18	0.0003

*Adapted, revised, and used with permission from Kim SC et al: Using the intraoperative hand held probe without lymphoscintigraphy or using only dye correlates with higher sensory morbidity following sentinel lymph node biopsy in breast cancer: A review of the literature. *World J Surg Oncol *2005, 3:64. [41].

^#^Statistics used to generate the p value: Fisher's exact test (2-tailed). A result was considered to be significant only if the p-value was lower than 0.05.

**Table 2:** Updated comparisons of average chronic pain and numbness/paresthesia morbidity between LS groups (+) performing lymphoscintigraphy and non LS groups (-) not performing lymphoscintigraphy in patients undergoing SLNB using radiotracer or using only dye. In general, studies using lymphoscintigraphy continue to have much lower levels of chronic sensory morbidity. Updated data by incorporation of four new references [21,22,23,24].

	Lymphoscintigraphy (+) Performed			Lymphoscintigraphy (-) Not Performed			
		
*Morbidity (Mor)	Mor (%)	Total Pt (N)	References	Mor (%)	Total Pt (N)	References	^#^p-value
Pain (>9m)	14.32%	1508	1,2,4,9,10,11,14,20,22,24	28.67%	143	6	< 0.0001
Numbness/Paresthesia (>9m)	9.22%	1052	1,4,9,11,13,14,17,20,23,24	23.17%	315	3,6,18,21^t^	< 0.0001

*Adapted, revised, updated and used with permission from Kim SC et al: Using the intraoperative hand held probe without lymphoscintigraphy or using only dye correlates with higher sensory morbidity following sentinel lymph node biopsy in breast cancer: A review of the literature. *World J Surg Oncol *2005, 3:64. [41].

^t^Use of LS is variable/suboptimal [21]. See text for details.

^#^Statistics used to generate the p value: Fisher's exact test (2-tailed). A result was considered to be significant only if the p-value was lower than 0.05.

**Table 3:** 

***Potential advantages of optimized lymphoscintigraphy ***
- Reduces morbidity compared to probe only method.
- Reduces short and long term costs of treatment resulting from morbidity.
- Facilitates minimal invasiveness, improving cosmetic results.
- Assists surgeons in planning their approach and harvesting the SN.
- Provides additional guidance for surgeons who are learning the SLNB technique.
- Reduces overall surgical costs (anesthesia time, operating room utilization) by shortening surgery.
- Provides a wide field of view survey covering multiple lymph node basins simultaneously improving staging.
- Diffusion fields emanating from injection sites are defined and assessed for partly hidden nodes by scaling.
- The effects of breast displacement maneuvers are easily assessed.
- Delineates multiple SN nodes, their position and intensity along lymphatic channels, time of appearance.
- Delineates intervening nodes in unexpected positions when prominent lymphatic channels are present.
- Estimates the position of the SNs in the body from triangulated body marking (TBM).
- Surface contamination and other quality control issues are easy to detect and implement.
- Dynamic imaging is possible and its potential benefits in select cases.
- Assesses the intensity of the SN for next day surgery and determines the need for additional injections.
- Alerts to a failed node visualization (tumor replacement) and the possibility of a more extensive ALND.
- Delineates reverse echelon nodes, persistent lymphatic pools/dilations, end-on effects.
- Guides the planning of radiation ports with the inclusion of internal mammary chains when present.
- Sitting views can resolve clumped nodes, not possible with the probe after anesthesia.
- Improved localizing performance in obese patients compared to the probe before incision.
- Reduces the chance of un-harvested SNs (false negatives) through comparisons (numerical/positional/intensity) with images.

***Potential disadvantages of lymphoscintigraphy ***

- Additional cost of procedure.
- Technically effort-intensive to fully optimize.
- Can delay surgery if not scheduled appropriately.
- Additional patient time required and any associated discomfort during imaging.
- Inadequate reimbursement for those performing it.

### Advantages of lymphoscintigraphy

#### Wide field of view

Optimized lymphoscintigraphy provides detailed, comprehensive information on sentinel node (SN) location and on drainage routes. It has the advantage of simultaneously sampling the entire chest for minutes at a time, in contrast to the probe, which samples only one particular point for a short time [38,42-45]. Given this enhanced global sensitivity, the camera is superior in the initial survey. In addition, the images allow identification of internal mammary and other extra axillary nodes [46-48] and rare cases of drainage to the contralateral axilla [49-53]. When SLNB is performed in patients with previous breast implant augmentation (where complex patterns of activity can arise), or in cases of previous SLNB, the images can prove invaluable [54-57].

#### Unique drainage patterns visualized

In melanoma, lymphoscintigraphy is accepted because globally unique drainage patterns exist. Information from the images potentially improves staging by mapping out drainage basins [58,59]. This advantage can be applied to the axilla in breast cancer patients, but on a smaller, finer scale. Even limited to the region of the axilla, lymphoscintigraphy can provide similar, detailed information, as the axilla contains multiple nodes at various levels.

#### Triangulated body markings improve precision

Utilizing lymphoscintigraphy, an accurate initial incision location and dissection route can be chosen in advance. When the patient's skin is marked with triangulation points during lymphoscintigraphy, the surgeon can place the incision optimally. The triangulation marks result in a 3-Dimensional pattern, which can help to direct the surgeon to the location of the nodes, especially in obese patients or those with faint SNs [38,40,43], (Figure 2). Consequently, the initial incision location and dissection route can be better planned for the best cosmetics and least tissue disruption. This is in contrast to an approach using only the probe without surface markings, which might be equivocal during the initial survey in some patients.

#### Invasiveness can be further minimized

Clinical practice reveals a clear trend for minimally invasive techniques and breast conservation during both initial diagnosis, staging and subsequent surgical treatment [60-66]. The additional localizing information provided by optimized lymphoscintigraphy will allow for fine tuning of the axillary surgical approach in many patients, further optimizing morbidity reduction.

#### Additional views (standing/sitting) are possible

The standing/sitting views that are possible with lymphoscintigraphy further improve accuracy. These views reveal adjacent nodes hidden by the injection site scatter. They also resolve "clumping" of sentinel nodes that can occur in the axilla in the supine patient while also eliminating negative "end on effects" of lymphatic channels which will be discussed below [38,43,67-69]. This is important in delineating the true number of radioactive nodes, and also informs the surgeons of what to expect, and how many nodes to potentially remove [70,71]. The standing position obviously can not be performed with the probe at the initial survey before incision once anesthesia is administered.

### Optimizing techniques

#### Injection technique and Hot nodes

Injection technique is an important factor contributing to the ease of finding the SN and the success of SLNB. Areolar-cutaneous junction injections and similar injections under the nipple increase SN activity, making the SN easier to find with the probe while generating optimal images [38,43,72]. These injections are very efficient in delivering activity to the SN, more so than perilesional or even intra/sub-dermal injections [72]. This is particularly important in the obese patient, where fat attenuates radioactivity and also increases distances between the SN and probe. In the setting of 15 cm of fat, less than 20% of the signal is left after attenuation. Increased distance further weakens the signal by 1/d^2^ (d = distance from probe to node) and directionality suffers [42,73]. Gamma camera sensitivity, conversely, does not appreciably change over distance [73].

Augmenting activity in the SN also facilitates next day surgery protocols [11,38,43,72,74-76]. Eighteen hours after injection, the ^99m^Tc radiotracer has decayed to where only 12.5% of the original activity remains in the SN [73]. Performing injections and obtaining images using protocols that augment SN activity on the day before surgery, will alleviate surgical scheduling issues/delays. It can also save operating room time, by avoiding potential delays caused by starting the technique in the morning. These advantages will result in a cost savings [74-76].

#### Role of blue dye

When fully optimized lymphoscintigraphy with hybrid combination radiotracer injections is performed [38,43,72,77], it is no longer necessary to utilize blue dye as a primary method of finding the SN. In this setting, blue dye serves primarily as a backup (in the rare cases when a radioactive node is not detected) or as a secondary method to find the SN. Dye also serves as a potential visual guide when probe directionality is occasionally poor. Overall, dye provides less benefit than radiotracer as noted in several studies [78-80].

An exclusively blue dye technique can be viewed as not fully fulfilling the primary goal of morbidity reduction that SLNB promises. It necessitates more extensive dissection, as the lymphatic ducts leading to nodes are exposed until the SNs are found. In comparison, probe guided SN extraction can variably detect the SN directly through tissues, and further guidance is provided by lymphoscintigraphy images and skin markings [81-83].

Some early studies have shown 19.7% to 32.2% of SNs detected by dye alone in patients where radiotracer was also used [84,85]. However, these studies utilized inefficient perilesional injection techniques and no imaging methods (probe guided only).

In contrast, King et al. used dermal injections of radiocolloid employing lymphoscintigraphy and perilesional injections of dye. It was demonstrated that in 1719 procedures, only 1.9% of all the SNs were blue-only, and did not contain radioactivity detected by probe [86]. In a subgroup of procedures where smaller volumes of dye were used (0.1 ml–1.0 ml), only 1.3% of SNs were identified by blue dye alone [86]. The rate of blue only SNs positive for disease was higher however, at 10.5%. This may reflect the lack of simultaneous perilesional and areolar radiotracer injections as part of a hybrid injection technique as suggested by our group, as only 85.8% of studies demonstrated nodes on the images [72,86]. With experience in using radiotracer, use of dye becomes less relevant as was demonstrated in a study of 500 patients by Derossis et al. where a SN identified only with dye and containing disease was seen in only 2% of cases [87].

In a recent study, Degnim et al. [88] report on 418 cases in which radiotracer and dye were concurrently administered for SLNB. In 380 of these, SNs were identified on the lymphoscintigraphy images, and were recovered with the probe and/or by visual guidance from the blue dye. In only 3 of these 380 cases (0.79%) was disease that altered patient staging found in a node that contained blue dye but did not contain radioactivity [88]. In other words, had dye not been used, disease that changed staging would have been missed in 0.79% of total cases where SNs were identified on lymphoscintigraphy [88]. It is possible that technical issues, i.e., suboptimal SN intensity resulting from using mainly perilesional and dermal over the tumor injections, played a role in these findings. SNs were demonstrated on lymphoscintigraphy images in only 90.9% (380/418) of total cases administered radiotracer and dye. This is generally below the expected SN lymphoscintigraphy visualization rate obtained with current optimal areolar radiotracer injection methods and camera imaging protocols. Optimal delivery of radiotracer to SNs is an important issue, because the SNs involved with tumor may have limited numbers of macrophages, which can make the nodes appear faint if the delivery of radiotracer is suboptimal [38,40,72,74]. Higher efficiency areolar injections will generally increase activity in SNs proportionally. This will reduce occurrences of faint, disease containing nodes that are below the threshold of detection by the probe and camera, resulting in these nodes being missed by these methods, but visualized with dye. Thus, it can be postulated that even lower rates of blue node only disease would have been found if more efficient methods of radiotracer injection and imaging were employed in this study. This needs to be formally investigated.

In a study utilizing a variant of our previously reported hybrid combination injections of dermal and perilesional radiocolloid [77,89], without using any blue dye injections at all, Freezer et al. demonstrated a total 2% false negative rate for SLNB in cases that received ALND who had dynamic lymphoscintigraphy with triangulated skin markings [90].

Lastly, King et al. [86] also reported on 143 patients undergoing prophylactic mastectomy and SN biopsy. Of these patients, 9.1% had occult carcinoma identified, and the SN was positive in only 1.4% (2/143). In these particular low risk patients, the estimated probability of a blue-only SN not containing any radioactivity that would alter patient stage is a very small fraction of that 1.4%. Thus the use of dye in any capacity in this limited context that may lead to additional dissection can certainly be questioned.

Allergic/anaphylactic reactions, tattooing of skin with surface injections and localized tissue inflammation have been reported as complications to dye [86,90,91].

### Examination of arguments against the use of lymphoscintigraphy

Several articles on lymphoscintigraphy question the value of imaging, and report no significant difference in the number of SNs found with and without images [37,92,93]. However, it is important to note that both injection and imaging techniques were key factors in these reports. Universally, less effective perilesional injections and non-optimized imaging techniques were utilized.

McMasters et al. reported that only 56% of patients who had lymphoscintigraphy performed showed axillary nodes. In addition, 36.2% showed no drainage to any basin at all on the lymphoscintigraphy images [92]. However, these results were certainly influenced by technique. The study was multi-center, and was performed in 1997–1999, with only delayed views after 45–60 minutes (no dynamic images). Only perilesional injections were utilized, not the currently preferred areolar or dermal injections [92]. Considering the variation in quality of the lymphoscintigraphy procedures performed at the different centers, this study cannot accurately reflect what is possible with optimized techniques of imaging, injection and triangulated patient body marking [38,43-45,72,94-96]. In fact, with current techniques, rates of visualization on lymphoscintigraphy are 97%–100% [11,94-96].

In a study by Burak et al. the authors conclude that routine preoperative lymphoscintigraphy is not necessary. However, in this study, image quality was severely compromised, and sentinel nodes were noted on the images in only 70.8% of patients. In addition to poor image quality, this study was based on a relatively small sample size: 24 patients, (figure 1) [37]. Furthermore, imaging was performed utilizing a lead shielding technique, and the results were very poor when the tumors were in the upper outer quadrant.

In a larger, Department of Defense study by DuPont et al. involving 516 patients who had imaging in 1997–1999, only 65% of patients demonstrated axillary nodes during lymphoscintigraphy [93]. However, here technical aspects again affected the findings. In this study, a suboptimally narrow energy window of 10% was used as opposed to an upwardly offset 16%–18% energy window which is recommended [38,43,73,96]. Again, only perilesional injections were used, and shielding of the injection site was also employed. The reported methods of Burak et al. and DuPont et al. also suggest that it is likely that suboptimal collimators were used, along with un-optimized image acquisition energy settings [38,43,73,96,97]. These technical shortcomings can be redressed by using high quality cast (non-foil) collimators and optimal camera energy settings, which completely obviate the need for lead shielding. Lead shielding complicates lymphoscintigraphy imaging greatly, and can lead to artifacts, missed sentinel nodes and false nodes [38,96,97].

Clearly, these studies were influenced by significant technical limitations, as evidenced by the described methods and/or poor quality sample images presented (figure 1). Furthermore, suboptimal injection technique also contributed to the findings, in that the more efficient injection methods (dermal or areolar) were not utilized. Unfortunately, these articles [37,92,93] still continue to be cited as proof of the lack of usefulness of lymphoscintigraphy [98,99]. Most importantly, none of them address the issues of potential morbidity reduction that the newer imaging techniques provide, focusing instead mainly on the SN detection rate or false negative rate [37,40,41,92,93].

### A new look at the literature on morbidity reduction with lymphoscintigraphy

Unfortunately, to date, no landmark study has been performed that directly compares the additional reduction in morbidity achieved by properly performed lymphoscintigraphy, (with optimal injection technique and triangulated patient body markings) vs. use of the probe alone or dye alone. In the absence of such a study, we recently performed a focused review of the literature, which indirectly sheds light on this question [41].

Kim SC et al. [41] reviewed 20 articles addressing the differences in morbidity between SLNB and ALND. Of these 20 articles, the authors identified 10 articles in which lymphoscintigraphy was used that were suitable for comparison to the 3 articles identified in which lymphoscintigraphy was not utilized [41].

The percentage of patients experiencing chronic sensory morbidity after SLNB in Kim's analysis of these articles are depicted in Table 1. It is very important to note that multiple confounding issues may exist when comparing morbidity findings among different studies. However, at the very least, a clear trend seems to be present in Kim's analysis, which reveals that nearly twice the chronic sensory morbidity was reported from SLNB in the articles not utilizing lymphoscintigraphy but using only the probe or using only dye, vs. those articles using both lymphoscintigraphy and the probe [41], [Table 1].

Subsequent to Kim's analysis, Purushotham AD et al. [21] reported on the results of a randomized controlled clinical trial conducted in patients with primary breast cancer, which sought to compare morbidity associated with SLNB and ALND. Three hospitals participated in this study, in which patients were randomly assigned to ALND or SLNB. The SLNB procedure included radiotracer and blue dye. Personal communication with authors of this study revealed the following: in all 3 hospitals, in the SLNB arm, in the majority of cases, lymphoscintigraphy was not performed and/or if performed, was done suboptimally. In one hospital, the use of lymphoscintigraphy was abandoned half way through the study due to a perceived lack of usefulness. In another hospital lymphoscintigraphy was not used in the vast majority of cases. In the third hospital, lymphoscintigraphy was used only because it was required by the clinical protocols of that hospital (a surgical teaching hospital), however, the imaging specialist's reports concerning the results of lymphoscintigraphy were not communicated to the surgeons prior to surgery [21]. In addition, at this hospital, SNs were not marked on the patient's bodies, thus the opportunity to utilize triangulated body marking, which provides surgeons with a reference for SN location in the body, was missed [21,38,40,44,45,57].

#### Updated evaluation of the literature

In the vast majority of the cases in the study by Purushotham AD et al., lymphoscintigraphy was either used suboptimally or not at all [21]. Therefore, for the purposes of comparative analysis to other studies, it can be considered to be a non-lymphoscintigraphy study. Purushotham's study (non lymphoscintigraphy) [21], along with three new studies in which lymphoscintigraphy was used [22-24], was subjected to the inclusion/exclusion criteria utilized by Kim et al. [41] in their previous analysis, and the data from these four new studies was integrated into Kim's previous data. A new analysis of the updated dataset was performed. The results are represented in Table 2.

This table shows that the incorporation of the new data confirms and strengthens the trend suggested in Kim's previous analysis [41]. The updated analysis demonstrates over twice the chronic sensory morbidity among the studies not using lymphoscintigraphy but using probe-only or dye-only (P < 0.0001.)

It is interesting to note that, in general, the authors of the articles with the highest long term sensory morbidity who did not use lymphoscintigraphy, or abandoned/discounted (as shown in Table 1 and 2 above) have also published the vast majority of articles questioning its overall value [3,6,18,21,37,92,98-105]. This may be due to their past experiences with lymphoscintigraphy, which have convinced them of its lack of utility. However, it is quite possible that the lymphoscintigraphy protocols that were utilized in the past by these authors may have been affected by serious technical issues (such as were described above), resulting in suboptimal images of limited utility. Furthermore, it is likely that triangulated body marking was not used to guide and reduce dissection and subsequent morbidity. Based on our extensive clinical experience, we are aware that there is clearly great variation in the quality and methods of lymphoscintigraphy being practiced [37-40].

#### Intraoperative injections vs. lymphoscintigraphy

Layeeque et al. propose injecting the ^99m^Tc sulfur colloid intra-operatively to eliminate the pain of injection, suggesting that vasovagal episodes and pain occur 10%–20% of the time with preoperative radiotracer injections [106]. In our experience pain can be well controlled by a combination of topical anesthetic applied to the skin and the simultaneous addition of anesthetic to the ^99m^Tc sulfur colloid syringe [38,43,72,77].

Layeeque et al. [106] also suggests that with intraoperative areolar radiotracer injections, lymphoscintigraphy can be avoided as a result of the improved efficiency of delivering radiocolloid to the SN provided by surface injections and by the rapid flow of tracer to the SN from the injection site, which can reach the sentinel node before the injection is completed [38,43,72,107]. Hotter nodes are easier to find with the probe, and there have been minor advances in probe design, promising slightly better directionality in future models [108]. However, these superficial injection techniques are accompanied by unique features that make the images obtained during lymphoscintigraphy, including delayed views, all the more important.

Performed during surgery or in the nuclear medicine department, areolar injections will produce very prominent lymphatic channels. These can complicate the removal of nodes due to the extreme activity that can be present in them. Immediately after areolar injection, a very dynamic process occurs. Prominent channels appear, often multiple, that often course a tortuous path [38,43-45,69,72,96,107,109-111]. Nodes can blend in with channels for over 30–120 minutes after injection. Additionally, channels can track superficially above the axillary SN before coursing internally and inferiorly to the SN, in an inverted J pattern [38,43,72]. At inflections in the channels, activity can appear as foci, since the observer is looking at times down/through the length of the channel as opposed to perpendicular to it (the end on effect) [38,43,69,77,107]. These end on effects are also noted in all regions of the breast. Dilations/ectasias, which appear immediately after injection, pose an additional problem for the surgeon as they represent pseudo sentinel nodes and can appear as distinct foci. These are much more common with the areolar injection techniques compared to perilesional injections, as much more activity over a shorter time period is concentrated in the lymphatic channels [38,43,72,107].

During the initial 30–120 minutes after intraoperative injection, the surgeon is faced with a constantly changing pattern of radioactivity. Pursuing what appear to be foci that in fact represent end on effects or pseudo sentinel nodes and not real nodes, can result in unnecessary dissection when using only the probe [38,43,69,72,77,107,111]. Because these complex patterns arise immediately after injection and the changes continue over time, the information the camera provides in the form of dynamic images, multi-angle views and delayed views, is valuable in resolving the true nature of the patterns, a process in which the probe is severely disadvantaged. In fact, intraoperative injections could actually prolong surgery and increase dissection/morbidity in some patients, as a result of the complex post injection dynamic patterns described above [38,43,45,55,68,69,72,74,96,106,107,110-112]. If internal mammary sentinel nodes are deemed important to visualize, then the use of concurrent perilesional injections as part of a hybrid injection protocol of areolar and perilesional injections is necessary, as areolar injections do not delineate internal mammary nodes to any extent [38,72,113-115]. Perilesional injections require much more time to visualize the sentinel nodes than areolar injections.

### Value of lymphoscintigraphy in special situations

There are a number of special situations in which information provided by lymphoscintigraphy is very valuable. These include situations of non-visualization of nodes, training, contamination, free pertechnetate, pregnancy and the elderly.

Even with the best areolar injection techniques, there are rare occasions when the nodes are not seen or only appear after an extended time period [72]. This can occur in older patients, obese patients or those with prior lumpectomies [43,72,77,110,116]. Knowing that the activity is weak or absent, an additional injection can be performed employing a higher dose and/or volume of radiotracer [40,72,112].

A final quality control check of the radiotracer occurs with imaging. This will readily show patterns of free pertechnetate, as well as surface contamination, which are more difficult to detect with the probe [38,40,77].

In situations where surgeons are in the initial steps of learning SLNB, a lack of lymphoscintigraphy images and triangulation reference points can be detrimental to the patient. SLNB is being performed with increasing frequency, fueled by demand from patients and prevailing trends. In the past SLNB was performed mostly by experienced investigators with a strong commitment to the technique. However, with rising demand, a greater number of less experienced mainstream surgeons are adopting SLNB (in some cases reluctantly), and performing the procedure. Here the lymphoscintigraphy images serve as a vital training tool, and can support those surgeons who are newly learning the technique of SLNB. Simulators have been also developed that can assist, along with the images, in training surgeons [117,118].

In pregnant women and in the elderly, SLNB is safe and accurate [119-121]. Since time spent under anesthesia in these patients should be minimized, knowing the number and location of sentinel nodes and lymphatic tracts before surgery will expedite SLNB removal, and accomplish the goal of minimizing anesthesia time.

#### Limiting the numbers of nodes removed

Using lymphoscintigraphy and triangulation, Kennedy et al. demonstrated that little benefit in additional sensitivity results from removing more than two sentinel nodes [122]. Similarly using lymphoscintigraphy, Schrenk et al. suggest that excising more than three nodes adds little to accuracy [123]. Identifying which node appears first and finding the location of the subsequent, more distant echelon nodes is even more important with areolar injections than perilesional injections. This is because areolar injections tend to delineate a greater number of echelon nodes, as a greater percentage of the injected activity enters more directly into the lymphatic channels and is available to spill down over to distant echelon nodes [38,40,43,72,74].

If faced with 6 hot nodes, it is crucial to know which nodes are more important to excise. Only the first two to four need to be excised if the sequence of appearance and position along the lymphatic chain is known, especially if both perilesional and areolar injections of radiotracer first drain to the same primary SN. Therefore, removal of all remote echelon nodes is probably not warranted in select patients with a very low probability of nodal disease. Surgeons not utilizing lymphoscintigraphy will be faced with several dilemmas: 1) not knowing which node was drained to by single or combination injections, 2) not knowing which node appeared first and second along the lymphatic channel, 3) not knowing the relative position of all the nodes along the lymphatic channel and 4) not knowing their relative intensities to each other before incision. They will need to consider taking all nodes out as the hottest node is not necessarily the one with disease [124,125]. In contrast, when lymphoscintigraphy has been performed, surgeons can approach the axilla in a more informed fashion. Furthermore, if only a single node is seen on the images, and clearly drained to by both perilesional and areolar injections, then extensive dissection for additional nodes can be avoided to minimize morbidity.

### Morbidity reduction is a central goal of SLNB

When nodes are "hot", any reasonably good surgeon can achieve good sensitivity with varying levels of dissection. The aim is to accomplish this with as little dissection as possible, and at the same time maintain or improve the sensitivity. Knowing in advance the total number, location and pattern of SNs and lymphatic channels will result in a reduction in the total time of surgery, anesthesia, operating room associated costs, in addition to improved sensitivity. Most importantly a more targeted surgical approach will result in a reduction in patient morbidity. We observed unique patterns of drainage that can have an impact on the false negative rate and morbidity [38,43,50,55,69-72,74,77,96,107,113,115]. Based on the vast experience in imaging of multiple authors as well as ourselves [11,38,40,42-45,47,48,50,55,57,69-72,74,77,96,107,111-115,126-129], the general techniques described here form the basis of a logical algorithm for patient management.

The fine details of optimizations for lymphoscintigraphy are extensive and relevant mainly to the imaging specialist. Factors associated with optimizing the procedure include collimator design, gamma camera energy windows, radiopharmaceutical preparation, pain control, injection location and technique, dynamic and multi-angle camera views, patient arm and body positions, triangulated body marking, breast displacement maneuvers, outlining techniques, image display and printing parameters and a thorough communication with the surgeon regarding the findings. A condensed, referenced protocol is presented in table 4.

**Table 4:** 

***Suggested optimized lymphoscintigraphy technique ***
***Camera/Outlining: ***
- High resolution low energy cast (non-foil) collimator [38,96,97].
- 128 × 128 matrix-dynamic, 256 × 256 matrix-static.
- Upwardly offset ^99m^Tc energy windows and separate ^57^Co energy windows (122 kev) [38,43,77,96].
- Decayed ^57^Co sheet source transmission outlining to limit exposure [38,96].

***Injection: ***
- Anesthetic cream (EMLA) applied to injection sites for 30+ minutes [38,43,77].
- Hybrid radiotracer injection technique: Concurrent perilesional (2–4 ml biased away from the axilla) and areolar-cutaneous "junction" injections "*LymphoBoost *" (LB), (0.2–1.0 ml). Total dose: 150–400+ uCi ^99m^Tc sulfur colloid for same day injections and surgery, 500–1000+ uCi for next day surgery. Higher LB volumes towards 1.0 ml tend to visualize nodes quicker and brighter but delineate more echelon nodes compared to lower volumes of 0.2 ml [38,40,43,72,74].
- High specific activity preparation, 100% filtered [130].
- Lidocain added to sulfur colloid syringe for additional pain control [38,43,77].
- Mild/short massage only [131].
- Deeper sub-lesional injections for internal mammary SN visualization if deemed important [113].
- Contamination control [77].

***Acquisition sequences: ***
- Optional post perilesional injection views.
- Dynamic lateral 100 frame 10 second images during areolar-cutaneous "junction" injection "*LymphoBoost" *(LB) [38,43,72,74].
- Optional immediate post dynamic early static sitting/standing views (see below).
- Delayed supine anterior and oblique 45° views with the arm out in the 90° surgical position and lateral views with the arm up towards the head with triangulated body marking of anterior and oblique 45° views.
- ^57^Co sheet source transmission outlining of anterior and lateral views [38,43,96].
- Sitting/Standing views highly recommended (see below).

***Additional optional maneuvers: ***
- Perform perilesional injection followed by 30 minute (or more) delayed views followed by LB injection (dynamic see above). Alternately delete perilesional injections altogether (only inject LB).
- *Adaptive Injection Technique *(AIT), re-inject different volumes of radiotracer based on imaging results [40],(data pending publication).
- Tape breast displacement for small breasts, for large/pendulous breasts use sitting views (see below) [38,43,96].
- Prone imaging [112], MOVA position [127], next day follow up views if two day.
- Avoid lead shielding the injection site [42,96,97].

***Triangulated body marking: ***
- See figure 2, [38,40,43].

***Sitting/Standing views: ***
- Highly recommended end of study anterior and lateral sitting/standing views with arm out in the 90° surgical position with chest pressed up against collimator (best resolution), two 1 minute frames each position to address motion if it occurs [38,40,43,67-71,96]. Works best in large breasted women.

***Display: ***
- Adjustment of upper level, gamma curve, pre-display low level data enhancement (pre-scale/contrast/threshold) and appropriate image summation [96].
- Viewing dynamic sequences in cine mode [107].

***Printing: ***
- Two sets of images for final supine views (marking views): with and without ^57^Co transmission scan (when performed) [38,96].
- Print images large enough for surgeons to clearly see anatomy. Optionally print sitting views and/or dynamic sequences if important [38,96,107].

***Reporting: ***
- Timely and detailed communications with surgeon before surgery to discuss findings, meaning/convention of markings and complex patterns. Number of SN based on supine and standing views, appearance sequence and perceived intensity, 3-D position in body, any extra-axillary or intramammary nodes, dilations/ectasias.

## Summary

SLNB has essentially become the standard of care irrespective of pending prospective data [132]. Besides the critical questions of false negative rates, equally important emphasis should be placed on further reducing morbidity by optimization of sentinel node excisional techniques. In order to accomplish this objective, SLNB methodology should include 1) detailed, optimized lymphoscintigraphy, 2) maneuvers to increase activity within the SN and 3) triangulated patient body marking. At the present time, in general, these methods are often either not used (alone or in combination), or if they are used, are done so in a suboptimal manner. A paradigm shift in departmental methods is needed to incorporate these valuable techniques, in order to meet the objectives of minimally invasive surgery, breast conservation and morbidity reduction. While it is may be true that all women who have SLNB do not benefit directly from lymphoscintigraphy images, in the patients where the images make a difference and reduce morbidity, a very meaningful improvement in patient care will have been achieved.

## Competing interests

The author(s) declare that they have no competing interests.

## Authors' contributions

**BRK **developed the initial concept and contributed to data analysis, design, revision and preparation of manuscript.

**MKS **contributed to data analysis, design, revision and preparation of manuscript.

**SCK **contributed to data analysis, design, revision and preparation of manuscript.

**DWK **contributed to data analysis

**AT **contributed to preparation, design and revision of manuscript.

**RMM **contributed to data analysis and preparation of manuscript.

**CK **contributed to data analysis, design, and preparation of manuscript.

## References

[B1] Schrenk P, Rieger R, Shamiyeh A, Wayand W (2000). Morbidity following sentinel lymph node biopsy versus axillary lymph node dissection for patients with breast carcinoma. Cancer.

[B2] Roumen RM, Kuijt GP, Liem IH, van Beek MW (2001). Treatment of 100 patients with sentinel node-negative breast cancer without further axillary dissection. Br J Surg.

[B3] Burak WE, Hollenbeck ST, Zervos EE, Hock KL, Kemp LC, Young DC (2002). Sentinel lymph node biopsy results in less postoperative morbidity compared with axillary lymph node dissection for breast cancer. Am J Surg.

[B4] Haid A, Koberle-Wuhrer R, Knauer M, Burtscher J, Fritzsche H, Peschina W, Jasarevic Z, Ammann M, Hergan K, Sturn H, Zimmermann G (2002). Morbidity of breast cancer patients following complete axillary dissection or sentinel node biopsy only: a comparative evaluation. Breast Cancer Res Treat.

[B5] Temple LK, Baron R, Cody HS, Fey JV, Thaler HT, Borgen PI, Heerdt AS, Montgomery LL, Petrek JA, Van Zee KJ (2002). Sensory morbidity after sentinel lymph node biopsy and axillary dissection: a prospective study of 233 women. Ann Surg Oncol.

[B6] Swenson KK, Nissen MJ, Ceronsky C, Swenson L, Lee MW, Tuttle TM (2002). Comparison of side effects between sentinel lymph node and axillary lymph node dissection for breast cancer. Ann Surg Oncol.

[B7] Haid A, Kuehn T, Konstantiniuk P, Koberle-Wuhrer R, Knauer M, Kreienberg R, Zimmermann G (2002). Shoulder-arm morbidity following axillary dissection and sentinel node only biopsy for breast cancer. Eur J Surg Oncol.

[B8] Leidenius M, Leppanen E, Krogerus L, von Smitten K (2003). Motion restriction and axillary web syndrome after sentinel node biopsy and axillary clearance in breast cancer. Am J Surg.

[B9] Schijven MP, Vingerhoets AJ, Rutten HJ, Nieuwenhuijzen GA, Roumen RM, van Bussel ME, Voogd AC (2003). Comparison of morbidity between axillary lymph node dissection and sentinel node biopsy. Eur J Surg Oncol.

[B10] Blanchard DK, Donohue JH, Reynolds C, Grant CS (2003). Relapse and morbidity in patients undergoing sentinel lymph node biopsy alone or with axillary dissection for breast cancer. Arch Surg.

[B11] Veronesi U, Paganelli G, Viale G, Luini A, Zurrida S, Galimberti V, Intra M, Veronesi P, Robertson C, Maisonneuve P, Renne G, De Cicco C, De Lucia F, Gennari R (2003). A randomized comparison of sentinel-node biopsy with routine axillary dissection in breast cancer. N Engl J Med.

[B12] Rietman JS, Dijkstra PU, Geertzen JH, Baas P, De Vries J, Dolsma W, Groothoff JW, Eisma WH, Hoekstra HJ (2003). Short-term morbidity of the upper limb after sentinel lymph node biopsy or axillary lymph node dissection for Stage I or II breast carcinoma. Cancer.

[B13] Peintinger F, Reitsamer R, Stranzl H, Ralph G (2003). Comparison of quality of life and arm complaints after axillary lymph node dissection vs sentinel lymph node biopsy in breast cancer patients. Br J Cancer.

[B14] Baron RH, Fey JV, Borgen PI, Van Zee KJ (2004). Eighteen sensations after breast cancer surgery: a two-year comparison of sentinel lymph node biopsy and axillary lymph node dissection. Oncol Nurs Forum.

[B15] Armer J, Fu MR, Wainstock JM, Zagar E, Jacobs LK (2004). Lymphedema following breast cancer treatment, including sentinel lymph node biopsy. Lymphology.

[B16] Ronka RH, Pamilo MS, von Smitten KA, Leidenius MH (2004). Breast lymphedema after breast conserving treatment. Acta Oncol.

[B17] Rietman JS, Dijkstra PU, Geertzen JH, Baas P, de Vries J, Dolsma WV, Groothoff JW, Eisma WH, Hoekstra HJ (2004). Treatment-related upper limb morbidity 1 year after sentinel lymph node biopsy or axillary lymph node dissection for stage I or II breast cancer. Ann Surg Oncol.

[B18] Langer S, Guenther JM, Haigh PI, Difronzo LA (2004). Lymphatic mapping improves staging and reduces morbidity in women undergoing total mastectomy for breast carcinoma. Am Surg.

[B19] Luini A, Gatti G, Zurrida S, Galimberti V, Paganelli G, Naninato P, Caldarella P, Rotmensz N, Winnikow E, Viale G (2005). The sentinel lymph node biopsy under local anesthesia in breast carcinoma: experience of the European Institute of Oncology and impact on quality of life. Breast Cancer Res Treat.

[B20] Ronka R, von Smitten K, Tasmuth T, Leidenius M (2005). One-year morbidity after sentinel node biopsy and breast surgery. Breast.

[B21] Purushotham AD, Upponi S, Klevesath MB, Bobrow L, Millar K, Myles JP, Duffy SW (2005). Morbidity after sentinel lymph node biopsy in primary breast cancer: results from a randomized controlled trial. J Clin Oncol.

[B22] Fleissig A, Fallowfield LJ, Langridge CI, Johnson L, Newcombe RG, Dixon JM, Kissin M, Mansel RE (2005). Post-operative arm morbidity and quality of life. Results of the ALMANAC randomised trial comparing sentinel node biopsy with standard axillary treatment in the management of patients with early breast cancer. Breast Cancer Res Treat.

[B23] Arnaud S, Houvenaeghel G, Moutardier V, Butarelli M, Martino M, Tallet A, Braud AC, Jacquemier J, Julian-Reynier C, Brenot-Rossi I (2004). Patients' and surgeons' perspectives on axillary surgery for breast cancer. Eur J Surg Oncol.

[B24] Barranger E, Dubernard G, Fleurence J, Antoine M, Darai E, Uzan S (2005). Subjective morbidity and quality of life after sentinel node biopsy and axillary lymph node dissection for breast cancer. J Surg Oncol.

[B25] Krag D, Ashikaga T (2003). The design of trials comparing sentinel-node surgery and axillary resection. N Engl J Med.

[B26] Badwe RA, Thorat MA, Parmar VV (2003). Sentinel-node biopsy in breast cancer [letter]. N Engl J Med.

[B27] De Salvo GL, Del Bianco P, Zavagno G (2003). Sentinel-node biopsy in breast cancer [letter]. N Engl J Med.

[B28] Munster AM (2003). Sentinel-node biopsy in breast cancer [letter]. N Engl J Med.

[B29] McMasters KM (2003). Sentinel-node biopsy in breast cancer [letter]. N Engl J Med.

[B30] Veronesi U, Maisonneuve P (2003). Sentinel-node biopsy in breast cancer [letter]. N Engl J Med.

[B31] Krag D, Ashikaga T (2003). Sentinel-node biopsy in breast cancer [letter]. N Engl J Med.

[B32] Singh Ranger G, Mokbel K (2003). The evolving role of sentinel lymph node biopsy for breast cancer. Eur J Surg Oncol.

[B33] Kern KA (2003). Achieving the lowest false-negative rate in peritumoral breast lymphatic mapping: the oncologic search for the Holy Grail. Ann Surg Oncol.

[B34] Cody HS (2003). Sentinel lymph node biopsy for breast cancer: does anybody not need one?. Ann Surg Oncol.

[B35] McMasters KM (2003). The eternally enigmatic axilla: further controversy about axillary lymph nodes in breast cancer. Ann Surg Oncol.

[B36] Rutgers EJ (2004). Sentinel node procedure in breast carcinoma: a valid tool to omit unnecessary axillary treatment or even more?. Eur J Cancer.

[B37] Burak WE, Walker MJ, Yee LD, Kim JA, Saha S, Hinkle G, Olsen JO, Pozderac R, Farrar WB (1999). Routine preoperative lymphoscintigraphy is not necessary prior to sentinel node biopsy for breast cancer. Am J Surg.

[B38] Krynyckyi BR, Kim CK, Goyenechea MR, Chan PT, Zhang ZY, Machac J (2004). Clinical breast lymphoscintigraphy: optimal techniques for performing studies, image atlas and analysis of images. Radiographics.

[B39] Pandey M, Deo SVS, Maharajan R (2005). Fallacies of preoperative lymphoscintigraphy in detecting sentinel node in breast cancer. World J Surg Oncol.

[B40] Krynyckyi BR, Kim SC, Kim CK (2005). Preoperative lymphoscintigraphy and triangulated patient body marking are important parts of the sentinel node process in breast cancer. World J Surg Oncol.

[B41] Kim SC, Kim DW, Moadel RM, Kim CK, Chatterjee S, Shafir MK, Travis A, Machac J, Krynyckyi BR (2005). Using the intraoperative hand held probe without lymphoscintigraphy or using only dye correlates with higher sensory morbidity following sentinel lymph node biopsy in breast cancer: A review of the literature. World J Surg Oncol.

[B42] Keshtgar MRS, Waddington WA, Lakhani SR, Ell PJ, Keshtgar MRS (1999). The Sentinel Node in Surgical Oncology.

[B43] Krynyckyi BR, Kim CK, Shafir MK, Mosci K, Machac J, Freeman LM (2003). Breast cancer and its management, the utility and technique of lymphoscintigraphy. Nuclear Medicine Annual; Philadelphia.

[B44] Mariani G, Moresco L, Viale G, Villa G, Bagnasco M, Canavese G, Buscombe J, Strauss HW, Paganelli G (2001). Radioguided sentinel lymph node biopsy in breast cancer surgery. J Nucl Med.

[B45] Mariani G, Erba P, Villa G, Gipponi M, Manca G, Boni G, Buffoni F, Castagnola F, Paganelli G, Strauss HW (2004). Lymphoscintigraphic and intraoperative detection of the sentinel lymph node in breast cancer patients: the nuclear medicine perspective. J Surg Oncol.

[B46] Bevilacqua JL, Gucciardo G, Cody HS, MacDonald KA, Sacchini V, Borgen PI, Van Zee KJ (2002). A selection algorithm for internal mammary sentinel lymph node biopsy in breast cancer. Eur J Surg Oncol.

[B47] Tanis PJ, Nieweg OE, Valdes Olmos RA, Peterse JL, Rutgers EJ, Hoefnagel CA, Kroon BB (2002). Impact of non-axillary sentinel node biopsy on staging and treatment of breast cancer patients. Br J Cancer.

[B48] Estourgie SH, Tanis PJ, Nieweg OE, Valdes Olmos RA, Rutgers EJ, Kroon BB (2003). Should the hunt for internal mammary chain sentinel nodes begin? An evaluation of 150 breast cancer patients. Ann Surg Oncol.

[B49] Allweis TM, Parson B, Klein M, Sklair-Levy M, Maly B, Rivkind A, Uziely B (2003). Breast cancer draining to bilateral axillary sentinel lymph nodes. Surgery.

[B50] Lim I, Shim J, Goyenechea M, Kim CK, Krynyckyi BR (2004). Drainage across midline to sentinel nodes in the contralateral axilla in breast cancer. Clin Nucl Med.

[B51] Barranger E, Montravers F, Kerrou K, Marpeau O, Raileanu I, Antoine M, Talbot JN, Uzan S (2004). Contralateral axillary sentinel lymph node drainage in breast cancer: A case report. J Surg Oncol.

[B52] Shimazu K, Tamaki Y, Taguchi T, Akazawa K, Inoue T, Noguchi S (2004). Sentinel lymph node biopsy using periareolar injection of radiocolloid for patients with neoadjuvant chemotherapy-treated breast carcinoma. Cancer.

[B53] Carmon M, Mintz A, Hain D, Olsha O (2005). Clinical implications of contralateral axillary sentinel lymph nodes. Breast.

[B54] Gray RJ, Forstner-Barthell AW, Pockaj BA, Schild SE, Halyard MY (2004). Breast-conserving therapy and sentinel lymph node biopsy are feasible in cancer patients with previous implant breast augmentation. Am J Surg.

[B55] Krynyckyi BR, Shim J, Goyenechea M, Kim CK (2004). Layering of activity around a breast implant capsule during lymphoscintigraphy. Clin Nucl Med.

[B56] Munhoz AM, Aldrighi C, Buschpiegel C, Ono C, Montag E, Fells K, Arruda E, Sturtz G, Kovac P, Filassi JR, Gemperli R, Ferreira MC (2005). The feasibility of sentinel lymph node detection in patients with previous transaxillary implant breast augmentation: preliminary results. Aesthetic Plast Surg.

[B57] Intra M, Trifiro G, Viale G, Rotmensz N, Gentilini OD, Soteldo J, Galimberti V, Veronesi P, Luini A, Paganelli G, Veronesi U (2005). Second biopsy of axillary sentinel lymph node for reappearing breast cancer after previous sentinel lymph node biopsy. Ann Surg Oncol.

[B58] Morris KT, Stevens JS, Pommier RF, Fletcher WS, Vetto JT (2001). Usefulness of preoperative lymphoscintigraphy for the identification of sentinel lymph nodes in melanoma. Am J Surg.

[B59] Uren RF, Howman-Giles R, Thompson JF (2003). Patterns of lymphatic drainage from the skin in patients with melanoma. J Nucl Med.

[B60] Paepke S, Schwarz-Boeger U, Kiechle M, Jacobs VR (2003). Axillary dissection with access minimized (ADAM): a new technique for lymph node dissection in conservative surgery for breast cancer. Int J Fertil Womens Med.

[B61] Ogawa Y, Ishikawa T, Sawada T, Chung SH, Osaka H, Takashima T, Onoda N, Kato Y, Ochi H, Hirakawa K (2003). Thoracoscopic internal mammary sentinel node biopsy for breast cancer. Surg Endosc.

[B62] Singletary SE, Dowlatshahi K, Dooley W, Hollenbeck ST, Kern K, Kuerer H, Newman LA, Simmons R, Whitworth P (2004). Minimally invasive operation for breast cancer. Curr Probl Surg.

[B63] Yamamoto D, Tanaka K (2004). A review of mammary ductoscopy in breast cancer. Breast J.

[B64] Veronesi U, Cascinelli N, Mariani L, Greco M, Saccozzi R, Luini A, Aguilar M, Marubini E (2002). Twenty-year follow-up of a randomized study comparing breast conserving surgery with radical mastectomy for early breast cancer. N Engl J Med.

[B65] Luini A, Zurrida S, Paganelli G, Galimberti V, Sacchini V, Monti S, Veronesi P, Viale G, Veronesi U (1999). Comparison of radioguided excision with wire localization of occult breast lesions. Br J Surg.

[B66] Krynyckyi BR, Shafir M, Zhang Z, Kim CK, Machac J (2004). TC-99M MAA improves on wire in localizing non-palpable breast lesions [abstract]. J Nuc Med.

[B67] Pierini A, Dworkin HJ (2001). Is the upright position more sensitive than the supine position in breast cancer sentinel node lymphoscintigraphy?. Clin Nucl Med.

[B68] Uren RF, Howman-Giles R, Renwick SB, Gillett D (2001). Lymphatic mapping of the breast: locating the sentinel lymph nodes. World J Surg.

[B69] Kim S, Youssef I, Kim CK, Machac J, Krynyckyi BR (2005). Prominent lymphatic channels simulating sentinel nodes: The utility of standing and delayed views in delineating the true number and position of nodes and the implications for further morbidity reduction. Clin Nucl Med.

[B70] Kim SH, Kim SC, Kim DW, Kim YJ, Youssef IM, Kim CK, Machac J, Krynyckyi BR (2005). Can different arm and body positions help in detecting more sentinel lymph nodes (SN) during lymphoscintigraphy (LS) [abstract]?. J Nucl Med.

[B71] Kim SH, Kim SC, Kim DW, Machac J, Kim CK, Krynyckyi BR (2005). The effect of different arm positions on sentinel node localization during lymphoscintigraphy [abstract]. J Nucl Med.

[B72] Krynyckyi BR, Kim CK, Mosci K, Fedorciw BJ, Zhang ZY, Lipszyc H, Machac J (2003). Areolar-cutaneous "junction" injections to augment sentinel node count activity. Clin Nucl Med.

[B73] Cherry SR, Sorenson JA, Phelps ME (2003). Physics in Nuclear Medicine.

[B74] Kim S, Kim CK, Krynyckyi BR (2005). Areolar-cutaneous junction injection boosts activity in sentinel node by more than 50 times compared to perilesional injection: implications for morbidity reduction. Am Surg.

[B75] Yeung HW, Cody III HS, Turlakow A, Riedel ER, Fey J, Gonen M, Nunez R, Yeh SD, Larson SM (2001). Lymphoscintigraphy and sentinel node localization in breast cancer patients: a comparison between 1-day and 2-day protocols. J Nucl Med.

[B76] Solorzano CC, Ross MI, Delpassand E, Mirza N, Akins JS, Kuerer HM, Meric F, Ames FC, Newman L, Feig B, Singletary SE, Hunt KK (2001). Utility of breast sentinel lymph node biopsy using day-before-surgery injection of high-dose 99mTc-labeled sulfur colloid. Ann Surg Oncol.

[B77] Krynyckyi BR, Miner M, Ragonese JM, Firestone M, Kim CK, Machac J (2000). Technical aspects of performing lymphoscintigraphy: Optimization of methods used to obtain images. Clin Nucl Med.

[B78] Kern KA (2002). Concordance and validation study of sentinel lymph node biopsy for breast cancer using subareolar injection of blue dye and technetium 99m sulfur colloid. J Am Coll Surg.

[B79] Lin KM, Patel TH, Ray A, Ota M, Jacobs L, Kuvshinoff B, Chung M, Watson M, Ota DM (2004). Intradermal radioisotope is superior to peritumoral blue dye or radioisotope in identifying breast cancer sentinel nodes. J Am Coll Surg.

[B80] Marrazzo A, Taormina P, Noto A, Cardinale G, Casa L, Mercadante S, Lo Gerfo D, David M (2004). Localization of the sentinel lymph node in breast cancer: prospective comparison of vital staining and radioactive tracing methods. Chir Ital.

[B81] Giuliano AE, Kirgan DM, Guenther JM, Morton DL (1994). Lymphatic mapping and sentinel lymphadenectomy for breast cancer. Ann Surg.

[B82] Kern KA (1999). Sentinel lymph node mapping in breast cancer using subareolar injection of blue dye. J Am Coll Surg.

[B83] Canavese G, Gipponi M, Catturich A, Di Somma C, Vecchio C, Rosato F, Tomei D, Cafiero F, Moresco L, Nicolo G, Carli F, Villa G, Buffoni F, Badellino F (1998). Sentinel lymph node mapping opens a new perspective in the surgical management of early-stage breast cancer: a combined approach with vital blue dye lymphatic mapping and radioguided surgery. Semin Surg Oncol.

[B84] Cox CE, Pendas S, Cox JM, Joseph E, Shons AR, Yeatman T, Ku NN, Lyman GH, Berman C, Haddad F, Reintgen DS (1998). Guidelines for sentinel node biopsy and lymphatic mapping of patients with breast cancer. Ann Surg.

[B85] Bass SS, Lyman GH, McCann CR, Ku NN, Berman C, Durand K, Bolano M, Cox S, Salud C, Reintgen DS, Cox CE (1999). Lymphatic mapping and sentinel lymph node biopsy. Breast J.

[B86] King TA, Fey JV, Van Zee KJ, Heerdt AS, Gemignani ML, Port ER, Sclafani L, Sacchini V, Petrek JA, Cody HS, Borgen PI, Montgomery LL (2004). A prospective analysis of the effect of blue-dye volume on sentinel lymph node mapping success and incidence of allergic reaction in patients with breast cancer. Ann Surg Oncol.

[B87] Derossis AM, Fey J, Yeung H, Yeh SD, Heerdt AS, Petrek J, VanZee KJ, Montgomery LL, Borgen PI, Cody HS (2001). A trend analysis of the relative value of blue dye and isotope localization in 2,000 consecutive cases of sentinel node biopsy for breast cancer. J Am Coll Surg.

[B88] Degnim AC, Oh K, Cimmino VM, Diehl KM, Chang AE, Newman LA, Sabel MS (2005). Is blue dye indicated for sentinel lymph node biopsy in breast cancer patients with a positive lymphoscintigram?. Ann Surg Oncol.

[B89] Krynyckyi BR, Firestone M, Eskandar Y, Kim CK, Machac J (2000). Dual method injection technique for breast lymphoscintigraphy to maximize visualization of sentinel nodes [abstract]. J Nuc Med.

[B90] Feezor RJ, Kasraeian A, Copeland EM, Schell SR, Hochwald SN, Cendan J, Drane W, Mastin S, Wilkinson E, Lind DS (2002). Sequential dermal-peritumoral radiocolloid injection for sentinel node biopsy for breast cancer: the University of Florida experience. Am Surg.

[B91] Singh-Ranger G, Mokbel K (2004). Capsular contraction following immediate reconstructive surgery for breast cancer – An association with methylene blue dye. Int Semin Surg Oncol.

[B92] McMasters KM, Wong SL, Tuttle TM, Carlson DJ, Brown CM, Dirk Noyes R, Glaser RL, Vennekotter DJ, Turk PS, Tate PS, Sardi A, Edwards MJ (2000). Preoperative lymphoscintigraphy for breast cancer does not improve the ability to identify axillary sentinel lymph nodes. Ann Surg.

[B93] Dupont EL, Kamath VJ, Ramnath EM, Shivers SC, Cox C, Berman C, Leight GS, Ross MI, Blumencranz P, Reintgen DS, DOD Breast Lymphatic Mapping Trial Investigators (2001). The role of lymphoscintigraphy in the management of the patient with breast cancer. Ann Surg Oncol.

[B94] Merson M, Fenaroli P, Gianatti A, Virotta G, Giuliano LG, Bonasegale A, Bambina S, Pericotti S, Guerra U, Tondini C (2004). Sentinel node biopsy in the surgical management of breast cancer: experience in a general hospital with a dedicated surgical team. Breast.

[B95] Trifiro G, Viale G, Gentilini O, Travaini LL, Paganelli G (2004). Sentinel node detection in pre-operative axillary staging. Eur J Nucl Med Mol Imaging.

[B96] Krynyckyi BR, Sata S, Zolty I, Kim CK, Knesaurek K (2004). Reducing exposure from ^57^Co sources during breast lymphoscintigraphy by optimizing energy windows and other suggested enhancements of acquisition and the display of images. J Nucl Med Technol.

[B97] Tsushima H, Yamanaga T, Shimonishi Y, Ochi H (2002). Usefulness of imaging method without using lead plate for sentinel lymph node scintigraphy. Kaku Igaku.

[B98] Kelley MC, Hansen N, McMasters KM (2004). Lymphatic mapping and sentinel lymphadenectomy for breast cancer. Am J Surg.

[B99] Tuttle TM (2004). Technical advances in sentinel lymph node biopsy for breast cancer. Am Surg.

[B100] Scoggins CR, Chagpar AB, Martin RC, McMasters KM (2005). Should sentinel lymph-node biopsy be used routinely for staging melanoma and breast cancers?. Nat Clin Pract Oncol.

[B101] Upponi SS, McIntosh SA, Wishart GC, Balan KK, Purushotham AD (2002). Sentinel lymph node biopsy in breast cancer – is lymphoscintigraphy really necessary?. Eur J Surg Oncol.

[B102] Chagpar AB, Kehdy F, Scoggins CR, Martin RC, Carlson DJ, Laidley AL, El-Eid SE, McGlothin TQ, Noyes RD, Ley PB, Tuttle TM, McMasters KM, University of Louisville Breast Sentinel Lymph Node Study (2005). Effect of lymphoscintigraphy drainage patterns on sentinel lymph node biopsy in patients with breast cancer. Am J Surg.

[B103] Guenther JM, Collins JC, Barnes G, O'Connell TX (2000). Selective lymphoscintigraphy: a necessary adjunct to dye-directed sentinel node biopsy for breast cancer?. Arch Surg.

[B104] McIntosh SA, Ravichandran D, Balan KK, Bobrow L, Wishart GC, Purushotham AD (2001). Sentinel lymph node biopsy in impalpable breast cancer. Breast.

[B105] Purushotham AD, Macmillan RD, Wishart GC (2005). Advances in axillary surgery for breast cancer-time for a tailored approach. Eur J Surg Oncol.

[B106] Layeeque R, Kepple J, Henry-Tillman RS, Adkins L, Kass R, Colvert M, Gibson R, Mancino A, Korourian S, Klimberg VS (2004). Intraoperative subareolar radioisotope injection for immediate sentinel lymph node biopsy. Ann Surg.

[B107] Kim SH, Shim J, Kim CK, Machac J, Krynyckyi BR (2004). Reverse echelon node and a lymphatic ectasia in the same patient during breast lymphoscintigraphy: The importance of injection and imaging technique. Br J Radiol.

[B108] Borgognoni L, Urso C, Vaggelli L, Brandani P, Gerlini G Sentinel node detection in melanoma patients using computer-assisted gamma probe with adjustable collimation and specifically designed forceps. Oral presentation, International Sentinel Node Congress, Los Angeles, CA, December 3–6, 2004.

[B109] Pelosi E, Bello M, Giors M, Ala A, Giani R, Bussone R, Bisi G (2004). Sentinel lymph node detection in patients with early-stage breast cancer: comparison of periareolar and subdermal/peritumoral injection techniques. J Nucl Med.

[B110] Kern KA (2001). Lymphoscintigraphic anatomy of sentinel lymphatic channels after subareolar injection of Technetium 99m sulfur colloid. J Am Coll Surg.

[B111] Uren RF, Thompson JF, Howman-Giles R, Sentinel nodes (2000). Interval nodes, lymphatic lakes, and accurate sentinel node identification. Clin Nucl Med.

[B112] Tanis PJ, van Sandick JW, Nieweg OE, Valdes Olmos RA, Rutgers EJ, Hoefnagel CA, Kroon BB (2002). The hidden sentinel node in breast cancer. Eur J Nucl Med Mol Imaging.

[B113] Krynyckyi BR, Chun H, Kim HH, Eskandar Y, Kim CK, Machac J (2003). Factors affecting visualization rates of internal mammary sentinel nodes during lymphoscintigraphy. J Nucl Med.

[B114] Estourgie SH, Nieweg OE, Olmos RA, Rutgers EJ, Kroon BB (2004). Lymphatic drainage patterns from the breast. Ann Surg.

[B115] Krynyckyi BR, Shim J, Kim CK (2004). Internal mammary chain drainage of breast cancer [letter]. Ann Surg.

[B116] Feldman SM, Krag DN, McNally RK, Moor BB, Weaver DL, Klein P (1999). Limitation in gamma probe localization of the sentinel node in breast cancer patients with large excisional biopsy. J Am Coll Surg.

[B117] Keshtgar MR, Chicken DW, Waddington WA, Raven W, Ell PJ (2005). A training simulator for sentinel node biopsy in breast cancer: a new standard. Eur J Surg Oncol.

[B118] Krynyckyi BR, Singh G, Colon D, Kim CK, Travis A, Kim SC, Machac J (2005). Letter to the editor [letter]. Eur J Surg Oncol.

[B119] Gentilini O, Cremonesi M, Trifiro G, Ferrari M, Baio SM, Caracciolo M, Rossi A, Smeets A, Galimberti V, Luini A, Tosi G, Paganelli G (2004). Safety of sentinel node biopsy in pregnant patients with breast cancer. Ann Oncol.

[B120] Keleher A, Wendt R, Delpassand E, Stachowiak AM, Kuerer HM (2004). The safety of lymphatic mapping in pregnant breast cancer patients using Tc-99m sulfur colloid. Breast J.

[B121] Gennari R, Rotmensz N, Perego E, Santos GD, Veronesi U (2004). Sentinel node biopsy in elderly breast cancer patients. Surg Oncol.

[B122] Kennedy RJ, Kollias J, Gill PG, Bochner M, Coventry BJ, Farshid G (2003). Removal of two sentinel nodes accurately stages the axilla in breast cancer. Br J Surg.

[B123] Schrenk P, Rehberger W, Shamiyeh A, Wayand W (2002). Sentinel node biopsy for breast cancer: does the number of sentinel nodes removed have an impact on the accuracy of finding a positive node?. J Surg Oncol.

[B124] McMasters KM, Wong SL, Chao C (2002). Comment on the article "Highest isotope count does not predict sentinel node positivity in all breast cancer patients," by Martin et al., August 2001, Annals of Surgical Oncology [letter]. Ann Surg Oncol.

[B125] Camp ER, Cendan JC, Feezor R, Lind DS, Wilkinson E, Copeland EM (2004). The hottest sentinel lymph node is not always the positive node. Am Surg.

[B126] Uren RF, Howman-Giles R, Chung D, Thompson JF (2004). Nuclear medicine aspects of melanoma and breast lymphatic mapping. Semin Oncol.

[B127] Haigh PI, Hansen NM, Giuliano AE, Edwards GK, Ye W, Glass EC (2000). Factors affecting sentinel node localization during preoperative breast lymphoscintigraphy. J Nucl Med.

[B128] Uren RF, Howman-Giles RB, Chung D, Thompson JF (2005). Role of lymphoscintigraphy for selective sentinel lymphadenectomy. Cancer Treat Res.

[B129] Thompson JF, Uren RF, Scolyer RA, Stretch JR (2005). Selective sentinel lymphadenectomy: progress to date and prospects for the future. Cancer Treat Res.

[B130] Krynyckyi BR, Zhang ZY, Kim CK, Lipszyc H, Mosci K, Machac J (2002). Effect of high specific-activity sulfur colloid preparations on sentinel node count rates. Clin Nucl Med.

[B131] Diaz NM, Vrcel V, Centeno BA, Muro-Cacho C (2005). Modes of benign mechanical transport of breast epithelial cells to axillary lymph nodes. Adv Anat Pathol.

[B132] Hampton T (2003). Surgeons "vote with their feet" for sentinel node biopsy for breast cancer staging. JAMA.

